# Congenital cardiac interventions during the peak phase of COVID-19 pandemics in the country in a pandemics hospital in Istanbul

**DOI:** 10.1017/S1047951120002000

**Published:** 2020-06-24

**Authors:** Murat Ugurlucan, Yahya Yildiz, Didem Melis Oztas, Senay Coban, Metin Onur Beyaz, Gizem Sari, Mustafa Ozer Ulukan, Atalay Karakaya, Binay Vatansever, Korhan Erkanli, Mert Meric, Orcun Unal, Demet Demirkol, Yilmaz Yozgat, Turkay Saritas, Abdullah Erdem, Celal Akdeniz, Halil Turkoglu

**Affiliations:** 1 Istanbul Medipol University, Faculty of Medicine, Department of Cardiovascular Surgery, Istanbul, Turkey; 2 Istanbul Medipol University, Faculty of Medicine, Department of Anesthesiology, Istanbul, Turkey; 3 Bagcilar Education and Research Hospital, Cardiovascular Surgery Clinic, Istanbul, Turkey; 4 Istanbul Medipol University, Faculty of Medicine, Department of Pediatric Cardiology, Istanbul, Turkey; 5 Istanbul Medipol University, Faculty of Medicine, Department of Pediatric Intensive Care, Istanbul, Turkey; 6 Istanbul University, Istanbul Medical Faculty, Department of Cardiovascular Surgery, Istanbul, Turkey; 7 Yedikule Chest Diseases and Chest Surgery Training and Research Hospital, Cardiovascular Surgery Clinic, Istanbul, Turkey; 8 Istanbul University, Istanbul Medical Faculty, Department of Pediatric Intensive Care, Istanbul, Turkey; 9 Bezm-i Alem Vakif Hospital, Department of Pediatric Cardiology, Istanbul, Turkey

**Keywords:** SARS-CoV-19, pandemics, congenital cardiac surgery, congenital cardiac percutaneous interventions

## Abstract

**Introduction:**

In this report, we aim to present our algorithm and results of patients with congenital cardiac disorders who underwent surgical or interventional procedures during the peak phase of the pandemics in our country.

**Patients and methods:**

The first COVID-19 case was diagnosed in Turkey on 11 March, 2020, and the peak phase seemed to end by the end of April. All the patients whom were referred, treated, or previously operated but still at the hospital during the peak phase of COVID-19 pandemics in the country were included into this retrospective study. Patient’s diagnosis, interventions, adverse events, and early post-procedural courses were studied.

**Results:**

Thirty-one patients with various diagnoses of congenital cardiovascular disorders were retrospectively reviewed. Ages of the patients ranged between 2 days and 16 years. Seventeen cases were males and 14 cases were females. Elective cases were postponed. Priority was given to interventional procedures, and five cases were treated percutaneously. Palliative procedures were preferred in patients whom presumably would require long hospital stay. Corrective procedures were not hesitated in prioritised stable patients. Mortality occurred in one patient. Eight patients out of 151 ICU admissions were diagnosed with COVID-19, and they were transferred to COVID-19 ICU immediately. Three nurses whom also took care of the paediatric cases became infected with SARS-CoV-2; however, the children did not catch the disease.

**Conclusion:**

Mandatory and emergent congenital cardiac percutaneous and surgical procedures may be performed with similar postoperative risks as there are no pandemics with meticulous care and preventive measures.

Since the first diagnosis of SARS-CoV-19 in China in December 2019, the disease has spread worldwide. It is accepted as an escalating pandemic by World Health Organization^1^ at the current stage affecting people at all age groups and leading to certain mortality and morbidity as well as tremendous occupancy rates in hospitals and ICUs.

As it is advised by worldwide national and international cardiology and cardiovascular surgery societies including our national society for cardiovascular surgery,^2^ it is a necessity to postpone elective cardiovascular procedures to the end of the pandemics in order to minimise the risk of transmission of the disease to an operated patient which may end up with the death of the case. It is also important to provide additional ICUs and ward beds for the patients infected with SARS-CoV-19 who require hospitalisation.

Patients with congenital cardiac defects account for a specific subgroup among patients with cardiovascular diseases, and indications for surgical and/or percutaneous interventions are diverse and versatile.^3^ Some of the cases are elective, whereas some require emergent procedures. Some has to be operated with robust timing; otherwise, they may lose the chance of ideal anatomical corrective option (e.g., patients with transposition of great arteries) or second-stage interventions (e.g., single ventricle cases). In addition, it is also possible to perform less invasive palliative and protective operations and postpone the main surgical treatment to a more appropriate stage to decrease the risk of the case (e.g., pulmonary bands for retarded patients with atrioventricular or ventricular septal defects, or shunt procedures for neonates with double ventricles and pulmonary stenosis or atresia). Moreover, with the increasing advances in technology and percutaneous techniques, many patients with congenital cardiac lesions also benefit from catheter-based treatment modalities.

There are no literature data regarding the results of congenital cardiac surgical or percutaneous interventions during the COVID-19 pandemics. In this report, we aim to present our institutional experiences with congenital cardiac patients during the COVID-19 pandemics.

## Patients and methods

The Ministry of Health officially declared the diagnosis of first COVID-19 case in Turkey by 11 March, 2020.^4^ Since then tremendous switch in the healthcare system and social security services was pronounced. Our hospital was announced among the healthcare providers during the pandemics as a “pandemics hospital,” and various alterations were executed for the care of the patients with COVID-19 disease. Although a separate building was provided for the care of these patients, unfortunately, it became rapidly full and the main building, including the floor of the cardiovascular surgery ward, as well as other wards were started to be occupied with SARS-CoV-19-infected patients.

The isolated ICU of the cardiovascular surgery branch with 18 beds with 4 isolated rooms spared for transplant cases became a general ICU. The other two high-volume ICUs of the hospital were reserved for the patients with the confirmed diagnosis of COVID-19, and the non-infected patients requiring intensive care were moved to cardiovascular surgery ICU. Unless confirmed with COVID-19, all the patients requiring intensive care and postoperative patients requiring ICU follow-up were allocated at the cardiovascular surgery ICU. If they are diagnosed with COVID-19 by nasopharyngeal swab, Polymerase Chain Reaction (PCR), or computerised tomography, they are transferred to one of the COVID-19 ICUs. The patients whom underwent cardiovascular surgery procedures were also followed at this ICU. Care was provided to all patients from different branches by the experienced constant staff of the cardiovascular surgery reanimation and nurse teams. Discharge from the ICU was adjusted between the reanimators and related branch physicians.

The children with congenital cardiac disorders whom required ICU follow-up following percutaneous or surgical interventions were also allocated in this originally cardiovascular surgery, however, former general ICU. Paediatric cardiac patients were either transferred to the new born ICU when they are extubated and completely weaned off inotropes or discharged from the hospital directly from this unit with seldom ward follow-up.

The patients whom were referred from other centres or admitted to our institution with the diagnosis or suspicion of congenital cardiac disorders during the peak period of the COVID-19 pandemics in the country or whom were previously operated; however, required hospitalisation after the first diagnosed case in the country on 11 March, 2020 was also included into this retrospective research. They were thoroughly investigated and evaluated by the paediatric cardiologists, paediatric cardiac surgeons, and anaesthesiologists and then the strategies were determined. All the patients coming from home without any infectious symptoms were either directly operated or percutaneously intervened. The cases with a history of hospitalisation longer than 24 hours were admitted to the isolation section of the ICU or refused if there were no available beds as a policy of the hospital. Patients were tested against COVID-19 and interventions were planned, unless emergent. The age, gender, body weight, diagnosis, history of previous interventions, the current procedures, duration of interventions, inotrope requirement, duration of mechanical ventilation, ICU and hospital stays, and adverse events were obtained from the institutional files. As a policy, all the studies regarding COVID-19 pandemics required special permission from Republic of Turkey, Ministry of Health. The study was approved by the Republic of Turkey, Ministry of Health. Further ethical approval was not obtained from the institutional ethics committee due to the retrospective nature of the research; however, the directors of the hospital were informed about the study.

## Results

There were 31 patients whom were referred or admitted to our institution with the diagnosis of congenital cardiac defects between 10 March and 30 April, 2020. As a consensus of the hospital, all the elective and stable cases were postponed by 15 March, 2020. Among the postponed cases, there was a female patient aged 8 months with the diagnosis of tetralogy of Fallot with normal growth, oxygen saturation above 90%, and without hypoxic spells. There were two girls at the age of 40 months and 44 months with the diagnosis of large ostium secundum-type atrial septal defect and superior-type secundum atrial septal defect with partial pulmonary venous return anomaly, respectively, with stable conditions and they were advised to re-admit for surgical treatment following COVID-19 pandemics, unless an emergency situation occurs. There was a single ventricle case in stable conditions and with oxygen saturation around 88% whom actually require bidirectional Gleen and his procedure was postponed. There was another boy aged 4 months with the diagnosis of perimembraneous ventricular septal defect sized 7 mm in the maximum diameter with normal growth and without any anticongestive therapy and without frequent pulmonary infections; and surgical treatment was also postponed for this particular case.

We were not able to accept a 10-day-old patient with the diagnosis of coarctation of aorta with depressed myocardial functions (ejection fraction: 40%–45%) whom was referred from another hospital, due to unavailability of isolation bed at our unit. We were informed that this baby was accepted by another hospital in the city for surgical treatment. The features of the patient whom were consulted but not treated at our institution are presented in Table 1.

**Table 1.** The demographic, interventional, operative, and postoperative features of the patients

Patient	Age (days)	Gender	Weight (kg)	Date of birth	Diagnosis	Percutaneous procedure	Surgical procedure	Ventilation time (hours)	ICU stay (days)	Hospital stay	Adverse events
1	240	F	–	–	TOF	–	Surgical treatment was postponed after COVID-19 pandemics, unless an emergency situation occurs	–	–	–	–
2	40 months	F	–	–	Large ostium secundum type atrial septal defect	–	Surgical treatment was postponed after COVID-19 pandemics, unless an emergency situation occurs	–	–	–	–
3	44 months	F	–	–	Superior type secundum atrial septal defect with partial pulmonary venous return anomaly	–	Surgical treatment was postponed after COVID-19 pandemics, unless an emergency situation occurs	–	–	–	–
4	7 months	M	–	–	Single ventricle with oxygen saturation 88%	–	Surgical treatment was postponed after COVID-19 pandemics, unless an emergency situation occurs	–	–	–	–
5	120	M	–	–	Perimembraneous ventricular septal defect	–	Surgical treatment was postponed after COVID-19 pandemics, unless an emergency situation occurs	–	–	–	–
6	10	M	3 kg	30/03/2020	AoCoA, bicuspid aortic valve, systolic dysfunction (EF: 40%–45%), PuHT	None	Not accepted due to unavailable isolation bed	–	–	–	–
7	312	F	6,7 kg	22/05/2019	Apical and perimembranous large VSDs with previous pulmonary band and occlusion of the right PA	Diagnostic (25/10/2019)	VSD closure, PA reconstruction (06/03/2020)	72 + 96 + 48	20	26	Antibiotic change. Multiple intibuations and extubations. Convulsions. Increased pulmonary secretions.
8	45	M	4.54 kg	24/01/2020	Double aortic arch	CT angiography (7/3/2020)	Division of the non-dominant arch (9/3/2020)	14	1	4	–
9	(16 years) 5760	F	50 kg	24/03/2004	Marfan syndrome-Anuloaortic ectasia Mitral valve prolapossus	None	Benthall-de Bono Procedure (10/03/2020)	7	2	7	–
10	240	F	8.2 kg	18/07/2019	TOF + additional VSD, right aortic arch	Diagnostic cardiac catheterisation	Total correction (11/03/2020)	24	2	5	–
11	(4 years) 1440	F	19 kg	03/09/2015	Double inlet, double outlet undetermined ventricle, pulmonary atresia with previous mBT shunt, and bidirectional Glenn (26/11/2018) Situs İnversus Totalis	None	Extracardiac fenestrated Fontan (12/03/2020)	48	2	13	–
12	7	M	2.9 kg	08/03/2020	TGA PDA	Balloon atrial septostomy (08/03/2020) (Z-5 Miller Balloon)	Arterial switch, VSD closure (18/03/2020)	72	25	25	Antibiotherapy change
13	(5 years) 1800	F	28 kg	31/12/2014	Congenital AV block, previous pacemaker implantation at the neonatal period – need for battery change	None	Pacemaker battery replacement (27/03/2020)	1	1	5	Delayed discharge due to delayed permission for intercity travel.
14	6	M	3.7 kg	25/03/2020	TGA, VSD (small) ASD PDA	None	Arterial switch, VSD closure (01/04/2020)	60	10	10	Antibiotherapy change
15	31	F	3.4 kg	06/03/2020	Unbalanced AVSD PDA Hypoplastic RV (mild) PuHT	None	PDA ligation, pulmonary band (07/04/2020)	48	8	8	Antibiotic change
16	28	M	4 kg	19/03/2020	DILV, DORV, hypoplastic RV, left AV valve hypoplasia, PuHT Secundum ASD, PDA	None	PDA ligation, pulmonary band (08/04/2020)	48	26	26	Antibiotherapy change
17	240	M	6 kg	17/08/2019	Intact ventricular septum with pulmonary atresia with previous mBT shunt (19/08/2019) and bidirectional Glenn (28/02/2020) – Occluded left PA	Percutaneous left PA dilatation (06/04/2020) (6 × 3 cmlik TYSHAK Balloon) – Unsuccessful	Off pump left pulmonary artery thromboendarterectomy, pericardial patch, left mBT shunt, right PA band (08/04/2020)	960 (40 days)	Still	Still	Caring nurse was diagnosed with SARS-CoV-2. The child did not catch COVID-19.
18	22	M	3.2 kg	25/03/2020	AoCoA, VSD (muscular), bicuspid aortic valve	None	AoCoa repair (extended resection, end to end anastomosis) (10/04/2020)	96	8	8	Antibiotic change
19	30	F	4 kg	17/03/2020	VSD (perimembraneous inlet, large-19X21 mm in size), additional apical VSD (5 mm in size) Hypoplastic RV Secundum ASD Persistent left SVC PuHT	None	PDA ligation, pulmonary band (13/04/2020)	48	7	7	Caring nurse was diagnosed with SARS-CoV-2. The child did not catch COVID-19.
20	6	F	3 kg	14/04/2020	TGA, VSD (small) ASD PDA	Balloon atrial septostomy (14/04/2020) (Z-5 Miller Balloon)	Arterial switch, VSD closure (20/04/2020)	48	9	9	Prolonged ICU stay due to antibiotherapy and rhythm follow up
21	83	F	3.4 kg	30/01/2020	Complete AVSD, annulary pancreas with previous abdominal surgery. Experienced sepsis attacks, in bad conditions	None	PDA ligation + pulmoner banding (22/04/2020)	Died on the 6th day while still intubated	Died on the 6th day while still at the ICU	Died on the 6th day	Sudden unexplained death. Antifungal and multiple antibiotics use
22	133	M	4.1 kg	17/12/2019	VSD	–	VSD closure (22/04/2020)	19	3	6	Caring nurse was diagnosed with SARS-CoV-2. The child did not catch COVID-19.
23	13	F	3.25 kg	15/04/2020	Supracardiac type TAPVR	–	TAPVR repair, no-touch technique (28/04/2020)	96	Still	Still	Delayed sternal closure. Antibiotic change.
24	86	M	4.6 kg	01/02/2020	AoCoa, ASD, VSD	Balloon angioplasty (11–02–2020)	AoCoa repair with patch (29/04/2020)	24	24	Still	–
25	1440 (4 years)	M	15.2 kg	12/07/2016	DORV, pulmonary atresia, situs inversus, rudimentary right ventricle with previous mBT shunt and atrial septectomy (27/07/2016), bidirectional Glenn (13/01/2017), right PA stent implantation (24/02/2017)	Diagnostic catheterisation + stent dilatation (01/05/2019)	Right PA reconstruction + central shunt (29/04/2020)	Still	Still	Still	–
26	101	M	3 kg	06/01/2020	Pulmonary stenosis	Pulmonary balloon valvuloplasty (18/03/2020) (6 × 2 cm peripheral balloon)	None	None	101	Still, due to bronchopulmonary displasia	–
27	(13 years) 4680	M	84 kg	02/05/2016	Stent implantation for AoCoA, device closure of the VSD (15/12/2012) – re-coarctation	Balloon angioplasty for AoCoA stent (18/03/2020) (14 × 3 cm TYHSAK Balloon)	None	None	0	1	–
28	2	M	2.8 kg	18/03/2020	VSD, pulmonary atresia, MAPCA	Diagnostic angiography (20/03/2020)	None (following due to SPO2>%85)	48	6	6	–
29	450	F	8 kg	16/01/2019	VSD	Transcatheter VSD closure (20/03/2020) (10 × 8 KONAR MF device)	None	None	0	1	–
30	180	M	4 kg	19/09/2019	TGA with VSD with VSD closure and arterial switch (02/12/2019) and RVOT reconstruction (15/01/2020) – inlet muscular VSD, apical 2–3 VSDs	Transcatheter inlet-muscular VSD closure (01/04/2020) (10 × 8 KONAR MF device)	None	Tracheostomised	Still	Still	Receiving multiple antibiotics
31	36	M	3.8 kg	07/03/2020	Complete AVSD, TOF, PDA	Palliative baloon valvulopasty (successful) (13/04/2020) (6 × 2 cm peripheral balloon)	None	24	1	3	–

* By 30 April, 2020

ASD: atrial septal defect; Ao CoA: aort coarctation; AV: atrioventricular; AVSD: atrioventricular septal defect; DILV: double inlet left ventricle; DORV: double outlet right ventricle; PA: pulmonary artery; PDA: patent ductus arteriosus; PuHT: pulmonary hypertension; RV: right ventricle; RVOT: right ventricle outflow tract; SVC: superior vena cava; TGA: transposition of great arteries; TOF: tetralogy of Fallot; VSD: ventricular septal defect; TAPVR: total anomalous pulmonary venous return.

In total, there were 25 patients whom received either surgical treatment or interventional procedure or both at our institution during the pandemics period in the country until the end of April. The demographic, interventional, operative, and postoperative features of the patients are summarised in Table 1.

Two patients were referred from another well-known institution. They had been operated, however, were on mechanical ventilator for 40 and 140 days, respectively. They received only supportive care at the cardiovascular surgery ICU of the institution; however, together with the COVID-19 adaptations of that university hospital, the paediatric cardiac cases were transferred to the paediatric ICU. Their primary physician was changed. Together with the change of their primary physician, better care and additional diagnostic measures to clarify the reason of not being able to be weaned off the ventilators were seeked. The patients were consulted with us. The diagnosis of the first case was intact ventricular septum with pulmonary atresia whom underwent left modified Blalock-Taussig shunt through left thoracotomy at the neonatal period followed by another modified Blalock-Taussig shunt between the brachiocephalic trunk and the main pulmonary artery (operation timing could not be identified). His last operation was a bidirectional Glenn procedure in February 2020. Investigations revealed increased Glenn pressure and occlusion of the left pulmonary artery. We attempted to percutaneously open the left pulmonary artery, however, could not be successful. The patient underwent surgical off pump left pulmonary artery thromboendarterectomy, left pulmonary artery reconstruction with a pericardial patch reaching till the left pulmonary hilus, a shunt from the brachiocephalic trunk to the left pulmonary artery, and right pulmonary artery banding. The surgical exploration of the mediastinum and the left pulmonary artery revealed extensive use of absorbable haemostatic materials over the pulmonary arteries, and lesser curvature of the aortic arch (which most probably led to pulmonary compression as well as unsuccessful pulmonary balloon angioplasty), undivided and not explored left modified Blalock-Taussig which was thought to be one of the facilitating reasons of left pulmonary occlusion due to intimal hyperplasia inside the graft and at the anastomosis line. The other patient had undergone arterial switch and ventricular septal defect closure procedures at the age of 10 days which was followed by right ventricular outflow tract reconstruction 44 days after the Jatene procedure. He was tracheostomised and on ventilator. Echocardiography at our institution revealed a 7 mm diameter inlet muscular ventricular septal defect with additional at least two 2–3 mm in diameter apical ventricular septal defects. The largest of the ventricular septal defect was closed percutaneously with a device. Both patients were transferred to the referring university hospital’s paediatric ICU. They are still followed at the paediatric ICU, require supportive ventilation therapy, receive antibiotherapy, and planned to be discharged as soon as optimal conditions are achieved.

There were three patients with transposition of great arteries. Two of the patients had very small inlet-type ventricular septal defects. Two of the patients underwent balloon atrial septostomy preoperatively. All the patients were operated on the first 10 days of their lives. Arterial switch operations together with ventricular and atrial septal defects closures were performed in all patients. The postoperative courses were uneventful. Patients could be extubated in 72 hours and were transferred to the new born ICU free of inotropic support and without the need for inotropes. These patients were discharged from the hospital 25, 10, and 9 days after surgery. All the patients required change in the antibiotherapy from cefazolin to piperacillin and tazobactam combination.

Three patients aged less around 1 month with the diagnosis of unbalanced atrioventricular septal defect, double inlet-double outlet single ventricle, and large perimembraneous (2 cm) and apical (1 cm) ventricular septal defects, respectively, underwent pulmonary banding operations with ductus ligation through left anterior thoracotomy. The patients weaned off the ventilator in 48 hours and were transferred to the new born ICU in good conditions. Two of the patients required antibiotherapy change to piperacillin and tazobactam combination. Another patient aged 83 days with the diagnosis of complete atrioventricular septal defect was at the hospital since birth. She had annulary pancreas and had abdominal surgery. She experienced sepsis attacks and alimentation problems. She received intravenous antibiotic and antifungal treatments and was in a critical condition. We performed patent ductus arteriosus ligation and pulmonary artery banding through left anterior thoracotomy in this case. Unfortunately, unexplained sudden death occurred in this patient on the 6th postoperative day.

One patient aged 16 days with the diagnosis of aortic coarctation underwent coarctation repair (extended resection and end-to-end anastomosis). The other patient was an 86-day-old male with the diagnosis of aortic coarctation and small atrial and ventricular septal defects with a history of aortic balloon angioplasty. He underwent aorta patchplasty, weaned off the ventilator in 6 hours, and transferred to the ward in 2 days. However, the ventilation time was 96 hours due to increased secretions for the first case and transferred to the new born ICU 5 days after the operation. We had to change the antibiotherapy of the case to ceftriaxone and to piperacillin and tazobactam combination.

A 6-month-old baby with large perimembraneous ventricular septal defect and systemic pulmonary hypertension with Down syndrome, growth retardation, and congestive heart failure despite anti-congestive therapy underwent ventricular septal defect closure. Ventilation time of the child was 24 hours, followed by 48 hours ICU stay, and 5 days hospitalisation.

A 60-month-old girl with congenital atrioventricular block whom received pacemaker implantation through left thoracotomy in whom the battery had been implanted over the left diaphragm at the neonatal period underwent battery exchange. The battery was removed from the left hemithorax and the new battery was implanted under the rectus fascia on the left abdominal wall. The patient was from another town, kept in the hospital and could be discharged from the hospital 5 days after as soon as a special permit for intercity transfer was received.

A 10-day-old girl with the diagnosis of supracardiac-type total anomalous pulmonary venous return underwent a corrective procedure. The sternum was left open for 48 hours and 2 days after sternal closure the patient could be extubated. She is still at the ICU at present in good condition. We needed to convert her antibiotherapy to meropenem and then added vancomycin.

A boy aged 3 years with single ventricle physiology whom had undergone a modified Blalock-Taussig shunt operation at the neonatal period followed by a bidirectional Glenn and stent implantation to the right pulmonary artery presented to the clinic with 57% oxygen saturation. The catheterisation indicated stenosed right pulmonary stent and extensive arteriovenous fistulae in the right lung. The heart team decision was right pulmonary reconstruction and right pulmonary shunt and the patient underwent removal of the pulmonary stent, pulmonary reconstruction with a pericardial patch, and central shunt from the ascending aorta to the right pulmonary artery. By 1st of May, the patient is still on mechanical ventilator, with oxygen saturation ranging between 60% and 70% despite 80%–90% FiO_2_ values.

At the very early periods of the disease in the country, there were relatively five elective patients. The first case had undetermined double inlet and outlet ventricle with pulmonary atresia whom underwent right modified Blalock-Taussig shunt followed by bidrectional Glenn procedure previously and underwent fenestrated Fontan procedure. Total correction (septal defect closure with right ventricular outflow tract reconstruction with pulmonary augmentation, i.e., monocusp transannular patch) was performed for the patient with tetralogy of Fallot. Another case was referred with the diagnosis of double aortic arch with feeding difficulties and dyspnoea and the patient underwent division of the non-dominant arch. The last case had large apical and perimembraneous ventricular septal defects with a history of pulmonary banding and the band occluding the right pulmonary artery underwent closure of the ventricular septal defects and pulmonary artery reconstruction. The last elective patient was a 16-year-old girl with Marfan syndrome with the diagnosis of anuloaortic ectasia whom was operated before the diagnosis of first COVID-19 case in the country, on 10 March, 2020. She underwent Benthall-deBono procedure. Either the ICU or the hospital stays of these patients exceeded the first diagnosis of COVID-19-positive patient in the country; thus, these patients were also added to the cohort. The single ventricle case was extubated in 6 hours. ICU stay was 2 days; however, the ward stay was complicated with alimentation and drainage complications and the patient could be discharged home after 13 days. The ventilation time and ICU stay for the patient with tetralogy of Fallot were 24 hours and 48 hours, respectively, and he was discharged home after 7 days. The patient with double aortic arch stayed 1 day at the ICU and discharged from the hospital in 4 days. The postoperative course of the patient with ventricular septal defects was complicated with rhythm problems, convulsions, and pulmonary infections leading to multiple intubations and extubation attempts and ICU stay lasted 18 days, but the patient could be discharged home on the postoperative 26th day. The Marfan syndrome patient stayed 1 night at the ICU and discharged home after 7 days.

Except the balloon atrial septostomy performed for the cases with transposition of great arteries, one postoperative case who received percutaneous ventricular septal closure and another patient with unsuccessful pulmonary dilatation, there were seven other patients whom underwent catheterisation procedures. Five patients underwent diagnostic or percutaneous interventional procedures. A 2-day-old baby with ventricular septal defect and pulmonary atresia received diagnostic catheterisation which revealed sufficient pulmonary circulation through major aortopulmonary collaterals and oxygen saturation above 85%. The decision for the particular case was followed up at least during the COVID-19 pandemics and the patient was discharged home. Two patients with pulmonary stenosis (a 34-day-old boy with tetralogy of Fallot with atrioventricular septal defect and a 72-day-old boy with isolated pulmonary stenosis) received successful balloon pulmonary valvuloplasty. A 14-month-old girl with the diagnosis of outlet muscular ventricular septal defect with growth retardation, dilated left heart chambers, and aortic insufficiency received transcatheter defect closure. A 156-month-old boy with previous stent implantation for the relief of aortic coarctation presented with uncontrolled hypertension and frequent headache attacks underwent percutaneous balloon dilatation of the stent. The isolated pulmonary stenosis case is still at the hospital due to bronchopulmonary dysplasia. The other pulmonary balloon case could be discharged from the ICU 3 days after the intervention. The remaining cases were discharged home the next day after catheterisation.

Since the patients were followed at the general ICU, the beds of the patients were next to the beds of the patients of general ICU. The same nurse had to take care of the adult patients and paediatric cases at the same time. Between the first diagnosis of the COVID-19 disease in Turkey and till the end of April 2020, there were 151 patients admitted to our ICU. Eight patients whom were formerly negative for COVID-19 were later on diagnosed with the disease and were immediately transferred to the COVID-19 ICU of the hospital. One head nurse and two other staff nurses were diagnosed with COVID-19 during the 2-month period and were sent home for medical treatment and 14 days quarantine. Two nurses returned back to work with confirmed negative tests after quarantine. All the nurses also took care of the children until their COVID-19 diagnosis. The patients whom received care by these staff were tested against COVID-19 as soon as their nurses were diagnosed COVID-19 positive; however, serial tests ended up with negative results in these children.

## Discussion

COVID-19 has become a serious health problem rapidly after its initial diagnosis in December 2019 in Wuhan, China. The disease is caused by SARS-CoV-2, a virus that belongs to corona virus family. It is highly contagious and has been obvious that by the end of April 2020, COVID-19 is evident all around the world infecting people in all nations. The World Health Organization has announced COVID-19 as pandemics by 11 March, 2020.^1^ It is probably the second pandemics after the H1N1 flu in 2009^5^; however, the most serious pandemics of the new century so far.

Unfortunately, the disease has not been taken into account very seriously despite warnings as a result of the lessons learned from increased mortality, morbidity, and hospitalisation rates from the initial experiences by many populations which ended up with a disaster with as high as 1000s of deaths every day and very sadly even inability to hospitalise many patients. Moreover, the economical power and development status of many countries have been insufficient to overcome the need for medical care of the patients despite converting many closed facilities including schools, dormitories, stadiums, or even the high-velocity trains into “mobile hospitals”.

As the number of cases starts to increase across our country, public health authorities strongly advised social distancing, self-isolation, and self-quarantine which were supported by the medical societies as well as social media. The government also announced certain precautions including curfew of elderly (above 60 years of age) and youngsters (below 20 years of age) at first. It was followed by national curfew staring at the weekends and followed by curfew at the weekends as well as certain other days to constrain COVID-19 and limit its transmission. However, nobody as in other countries^6^ is able to estimate accurately the extent to which COVID-19 will affect the population in terms of rates of incidence, duration, and recovery.^7^ On the other hand, the healthcare politics and strategies against the dissemination of COVID-19 or treatment of patients with COVID-19 seems to be successful so far with the decreasing number of cases by the end of April 2020 as well speculations through certain graphics indicating a possible end of the disease soon in our country. The novel corona virus has infected more than 200,000 people all around the world, affecting daily lives and changing the world into an economical burden.^8^ The pandemic also has changed the daily routine of doctors including cardiovascular surgeons through different ways.

Certain issues have to be addressed regarding the cardiovascular disorders during the course of the pandemics and COVID-19 disease. Although COVID-19 is primarily a disease of the lungs, lessons learnt through its course indicated at least 15% patients face with cardiovascular comorbidities^9^ ranging between increased incidence of venous thrombosis^10,11^ and myocarditis leading to decreased ejection fraction.^12^ In addition, the course of the disease is also more severe in patients with existing cardiovascular disorders. It may even be worse in a postoperative cardiac case, as the postoperative period is an inflammatory state and if the patient is infected with corona virus, the course of the pulmonic viral infection may be drammatic.^7^

The pandemic very shortly ended up in extreme shortage of available ICUs and ward beds, ventilators and other medical equipment, medications, blood and blood products, and also shortage of healthcare providers all around the world.^3,7^ Obviously, everything is spared for the care of COVID-19-positive cases requiring hospitalisation. An additional issue rose with the risk of infection of the healthcare providers and non-COVID-19 patients requiring hospitalisation. Eventually, many doctors, nurses, and other healthcare personnel got infected, many of them catching the disease most probably at the hospitals. Hence, it has been logic and ethical to postpone elective procedures which have to be decided by a team. Mavioglu et al^13^ developed a level of priority statement for adult cardiovascular procedures and concluded that cases should be considered on an individualised basis by a heart team. Because, not only the patients themselves are at risk of COVID-19 but also the family members would be at substantial risk.

Incidence of congenital cardiovascular disorders is approximately 1% and babies will continue to be born.^14^ Congenital cardiac surgery and cardiology are two dedication branches and have to be performed by highly skilled and well-educated physicians; otherwise, outcomes of the patients may be incomplete or disastrous. In addition to the capacity and capability of the physicians dealing with congenital cardiac disorders, they need to be backed up appropriately to save the lives of the children with congenital cardiac pathologies. Because of the current challenging pandemic status of the healthcare facilities, congenital cardiac procedures have to be adjusted wisely. Prioritisation, difficult decisions, and triage of patients have to be adjusted by highly specialised heart team composed of cardiac surgeons, cardiologists, and anaesthesiologists having experience with congenital cardiac pathologies. Luckily, unlike adults, the major problem has not been related to COVID-19 disease itself in the paediatric population; however, routine care of the children with CHDs has been compromised due to the restriction of the healthcare resources including available beds, ventilators, or healthcare providers solely for CHD patients. Even the paediatric cardiac surgeons are deployed for the care of the patients with COVID-19 temporarily.^3^

It was the same situation at our institution. We postponed all the elective patients with congenital cardiac pathologies and operated on only emergency cases or patients otherwise would lose the chance of corrective or secondary procedures such as patients with transposition of great arteries or patients with single ventricle physiologies. We preferred palliative procedures in patients whom presumed to have a long ICU stay, e.g., pulmonary band for a critically ill baby with atrioventricular septal defect or a borderline right ventricular volume in a patient with large ventricular septal defects. Additionally, the tendency was percutaneous procedures such as dilatation of the previously implanted stents, ductal and right ventricular outflow tract balloons, or stents instead of shunt procedures or device closure of septal defects.

The timing of surgery in CHDs is determined depending on patient-related factors as well as guidelines. The responsibility of the patient in such a pandemic situation should be taken with a heart team, and the pros and cons of the decision should be discussed with the families in details and with transparency.^3^ If possible, online or phone conversations rather than face to face at the hospital setting should be preferred. It is important to minimise the exposure of patients from outside visitors including families in case of a pandemic situation. Every visitor has the risks of carrying infection to the hospital as well as getting infected at the hospital environment especially when considering asymptomatic shedding of the truly infected people by the SARS-CoV-2. Social distancing and wearing face masks are crucial for the prevention of the transmission of airborne viral diseases.^3^ Although psychologically it is not easy for the families having their children receiving congenital cardiac surgery, the ICU visits of the babies were prohibited at our institution and breast milk is accepted which is prepared at home. The ward stays were as short as possible and only one parent is allowed with the child. In order to minimise the risk of being infected by increased exposure or because of allocation for the care of the COVID-19 cases, the training activities of the residents are also postponed. The cardiac surgeons or paediatric cardiac surgery residents may also play a crucial role during the extracorporeal membrane oxygenation support of the hypoxic COVID-19 cases.^3^

Testing of children and hospital admitants against COVID-19 should have an important influence during decision making for the paediatric cardiac patients.^3^ Isolation beds may also be preferred until the results of the tests from outside hospital admissions. Additionally, the procedures or even the decisions regarding the procedures of the paediatric cases may be postponed until the results of the tests. Otherwise, in case of emergency settings and especially patients with a history of COVID-19 exposure or symptoms of airway tract infection, it may be wise to perform the procedures in operating theatres spared only for COVID-19-positive cases.

All of our patients had to stay next to the adult patients from various specialties including general surgery, neurosurgery, orthopedics, etc. or patients requiring ICU follow-up in a COVID-19-negative ICU. We believe such kind of hospitalisation may have contributed to especially the additional antibiotherapy and lengthy hospital stays.

## Limitations

There are three major limitations of the study. First of all, it is a retrospective research. The second limitation is the small size of the cohort. When taking into account all the factors such as the first patient diagnosed in the country and the peak period until the end of April, together with preventive and economic measures to spare healthcare supplies for COVID-19 cases as well as strategy to postpone elective cases, we ended up with 25 cases. Two of our cases were referred from another university hospital paediatric ICU, and as soon as the patients are stabilised after their interventions, they were transferred to the referring centre for ICU follow-up in order to provide bed for the other patients requiring intervention at our institution. The same was also applied for the patients coming from the neonatal ICU of our institution. Otherwise, probably we would not be able to reach that number of cases due to the shortage of the ICU beds. The last limitation is the short postoperative follow-up period of the patients to the maximum of less than 2 months. Still some of our patients are at the hospitals or ICUs. Despite three of the nurses at our ICU were diagnosed with COVID-19, two have returned back to work after their treatment and quarantine. The patients whom received care from these nurses did not catch the disease confirmed with serial SARS-CoV-2 PCR presumably due to meticulous preventive measures such as routine use of masks. The lengthy stay of the cases or change in the antibiotic regimes may be due to other reasons including patient-related factors as well as mixed hospitalisation with patients from other medical divisions and chronic ICU patients.

## Conclusion

Birth is inevitable, and the percentage of congenital cardiac defects has not changed for long as being 1%.^14^ Unlike many branches, cardiac evaluation and required interventional procedures have to continue to save the lives of many patients with congenital cardiac disorders. Literature lacks a level of priority statement for the patients with congenital cardiac disorders; however, the decisions have to be made with a multidisciplinary paediatric heart team approach. Despite many preventive measures, either we or the patients will inevitably somehow catch SARS-CoV-2. Despite limited resources and risk of infection, it is still possible to conduct congenital cardiac procedures with meticulous care and physician protective equipments to prevent disease transmission to the patients.
